# Interventions to increase physical activity: An analysis of candidate behavioural mechanisms

**DOI:** 10.1016/j.pmedr.2022.101880

**Published:** 2022-07-04

**Authors:** Laura Gormley, Cameron A. Belton, Peter D. Lunn, Deirdre A. Robertson

**Affiliations:** a Behavioural Research Unit, Economic and Social Research Institute, Dublin, Ireland; b Institute of Education, Dublin City University, Ireland; c Department of Economics, Trinity College Dublin, Ireland; d School of Psychology, Trinity College Dublin, Ireland

**Keywords:** Behavioural science, Physical activity, Exercise, Disadvantage, Intervention

## Abstract

Physical inactivity is a significant driver of health and social inequalities, particularly affecting socioeconomically disadvantaged communities. This poses a major challenge to policymakers worldwide. Despite the large volume of original research and reviews that focus on the design and evaluation of interventions to increase physical activity, there remains little consensus on which interventions are likely to work. This paper discusses physical activity interventions through the lens of behavioural science. We consider the conclusions drawn by previous reviews of this literature and link them to potential behavioural mechanisms that might explain them. We categorise interventions into three broad types: physical environment, information provision and social context, and discuss specific components within each that are known to influence behaviour. The paper is not a systematic nor an exhaustive review. The recommendations are not for implementation without testing. Rather, the paper contributes an analysis of how existing evidence can be used to design research and interventions in future to test not just the main outcome, but the behavioural mechanisms that may determine success.

## Highlights

• The physical activity literature is large, diverse and often atheoretical.
• Behavioural science could help to inform physical activity interventions.
• Multi-level interventions are common but may incorporate conflicting components.
• Future designs should test individual mechanisms within multi-level interventions.

## 1 Introduction

Changing behaviour is difficult. Changing health behaviours, where immediate incentives for unhealthy behaviour often outweigh incentives for healthy behaviour, is even more so. This is particularly apparent for physical activity, where the interaction of personal, societal and environmental structures of modern society mean that 31% of adults are physically inactive, and a higher proportion in disadvantaged groups (Craike et al., 2018, Guthold et al., 2018, Hallal et al., 2012, Lunn, 2007). Physical inactivity is now the fourth leading risk factor for mortality worldwide (Kohl et al., 2012, World Health Organization, 2018).

A decade ago, a paper published in Preventive Medicine argued that behavioural economics could be harnessed to increase physical activity (Zimmerman, 2009). The field of behavioural economics has expanded rapidly since and behavioural science is regularly applied to policy (Matjasko et al., 2016). However, much physical activity research still rests on the rational choice model of behaviour, which assumes that once individuals are armed with information, their activity will increase (Kelly and Barker, 2016). Information campaigns are common but reviews conclude that they produce, at best, modest effects (Cleland et al., 2012, Datta and Mullainathan, 2014, Olstad et al., 2017). Consistent with this, behavioural science research shows that how individuals interact with their environments influences decisions in systematic ways, often overriding cost-benefit rationalisations (e.g. Luoto and Carman, 2014, Mitchell et al., 2013, Zimmerman, 2009).

Many lessons from behavioural science could potentially inform physical activity interventions, but an assessment of how reported interventions in the literature relate to behavioural mechanisms is not readily available. This paper aims to address the gap. The field of research on physical activity interventions is vast and diverse. It is so large that there now exist umbrella reviews of reviews on the literature (e.g. Craike et al., 2018). Reviews such as this make broad conclusions about what predicts a successful intervention, but these are not always granular enough to provide future directions for researchers or practitioners. The aim of this paper is not to draw its own conclusions from the literature, but to link existing conclusions to the behavioural mechanisms that might explain them. The intention is to gain a greater insight into why some interventions are more successful than others and to generate hypotheses for future interventions to be tested. The paper is not exhaustive or comprehensive and nor does it claim to make a definitive list of the behavioural mechanisms involved. Rather, it is a discussion piece about how behavioural science may explain some common findings, and how this might direct future research.

## 2 Methods

The paper was commissioned by a public agency and aims to provide lessons for policy. As such, the initial search was for interventions for socially disadvantaged groups, where need is greater.

Few intervention studies have based interventions on theories of behaviour, while those that have often lack detail about how theory was applied (Cleland et al., 2012, Craike et al., 2018). We link the conclusions drawn by previous reviews of the literature to behavioural mechanisms that might explain them. As conclusions from individual studies may not be representative of the whole field, we include only conclusions from review papers. We took a four-step approach to do this:

1) An umbrella review on physical activity interventions in socially disadvantaged groups had searched for literature up to May 2017 (Craike et al., 2018). As this literature is so large, we took this as a starting point and updated it to September 2021 using the same search terms. We only included studies on adults and not on children or adolescents. The original umbrella review cited 5 studies that made useful conclusions about what determines a successful physical activity intervention in disadvantaged adult populations. We added 6 new reviews based on our updated search and then 2 that were not carried out in disadvantaged populations but that discussed specific behavioural mechanisms. The relevant conclusions of the final 13 reviews included are listed in Table 1.

**Table 1:** Overview of summary statements from previous reviews, links to behavioural mechanisms and recommendations for future work.

Summary Statements	Possible Behavioural Mechanism	Recommendation
**Environment**		
Poor physical design is a barrier to physical activity but many interventions involving changes to built environments are ineffective (Durand et al., 2011, Kramer et al., 2017, Olstad et al., 2017).	The environment subtly but often substantially influences behaviour.	Environmental regeneration schemes should consider deliberately incorporating nudges into a design and testing these against control schemes.
Environmental changes alone are not effective without raising awareness of them or making them socially attractive (Hunter et al., 2019, Kramer et al., 2017).	Behavioural interventions are most successful when they make the intended change frictionless, attractive, social and timely.	Environmental regeneration schemes could test ways of removing all frictions, no matter how small, and drawing sufficient attention to the regeneration to make facilities attractive and socially acceptable to use.
How socially connected people feel to their environment is correlated with physical activity (Sawyer et al., 2017).	1. When people are more attached to their environment, they see it as safer and are more likely to look after it. 2. When people own something or have been involved in the creation of something, they value it more.	Environmental regeneration schemes could test whether actively involving communities in the regeneration and purposefully cultivating a sense of ownership and co-creation increases usage.

**Information provision**		
Interventions that focus on one behaviour or fewer techniques tend to be more successful than those focussing on multiple behaviours or techniques (Bull et al., 2018, Craike et al., 2018, Michie et al., 2009).	1. Goals can motivate behaviour changes but having too many goals at one time can be de-motivating and stressful. 2. When strong arguments for doing something are combined with weaker arguments for doing it, it reduces the effectiveness of the strong arguments (Presenter’s Paradox).	Information provision interventions could test whether giving people one strong reason for changing their behaviour, or having them generate their own strong reason, is more effective at increasing physical activity than giving many reasons.
1. Providing information on the antecedents of exercise can decrease effectiveness (Bull et al., 2018).2. Giving opportunity to practice exercise or providing feedback through pedometers can increase effectiveness (Bravata et al., 2007, Bull et al., 2018).	1. Feedback given too far in advance of a behaviour is not motivating. 2. When feedback is given immediately after a behaviour is carried out, it is motivating and increases likelihood of that behaviour being repeated.	Interventions that involve feedback from a practitioner could compare giving feedback prior to the intervention compared to during it. More work could be done on Just In Time Adaptive Interventions that are currently too underpowered to detect effects.
Information provision interventions are mostly ineffective but reports on the methods used are lacking in detail meaning analysis is difficult (Craike et al., 2018).	Information provision is not straightforward, its success may depend at least in part on how the information is framed.	Information provision interventions could test different ways of framing information such as making the goal gain-framed rather than loss-framed to assess whether this changes outcomes overall and/or differs by subgroups.

**Social Context**		
Group or community-focussed interventions are more effective than individually-targeted ones (Cleland et al., 2012, Cleland et al., 2013, Craike et al., 2018, Taylor et al., 1998).	An individual’s behaviour is influenced by what they think other people are doing.	Group-based interventions could test whether sharing information about the average levels of physical activity within the group during an intervention could help to change the social norm of inactivity and increase individuals’ own activity.
Interventions that promote group cohesiveness are most successful (Burke et al., 2006) (Note this is not a review of disadvantaged populations).	Individuals incorporate the values of a group they affiliate with into their own sense of self and align their attitudes and behaviours to it	Group-based interventions could test whether adding elements designed to build a team-like mentality during an intervention increases physical activity.
Incentives, particularly those that promote accountability if the behaviour is not achieved, can increase physical activity (Mitchell et al., 2013) (Note this is not a review of disadvantaged populations).	Accountability can influence behaviour as individuals may wish to benefit a group they are part of, save face, gain status or avoid the regret of not doing something they intended to.	Interventions involving non-financial forms of commitment contracts and incentives could be tested in socially disadvantaged groups.
2) We generated a list of the broad conclusions made by these reviews about what predicts successful physical activity interventions. We grouped these into three categories that summarise which aspects of an intervention they describe: the environment, information provision and social context (Table 1).
3) We compiled a list of potential behavioural mechanisms that may explain the reviews’ conclusions. These were based on our own expertise and on hypotheses from two other papers (Thorgeirsson and Kawachi, 2013, Zimmerman, 2009). These included norms, framing, habit formation, present bias, feedback, commitment contracts, loss aversion, channel factors, anchoring and status quo bias.
4) As little previous research concentrates on behavioural mechanisms, we searched for additional studies that had leveraged these and may have been missed by previous reviews. We searched for academic literature on PubMed and grey literature (policy documents, unpublished theses, non-academic papers) on OpenGrey.eu. As research examining these mechanisms in physical activity interventions is limited, we did not restrict this search to any one group.

The paper is not a systematic review of literature nor an exhaustive overview. Instead, it uses the conclusions from existing reviews of the literature to analyse the behavioural mechanisms that may explain them, coupled with examples. We intend this paper to be a launchpad from which to generate hypotheses about components for successful interventions that could be tested in future research. We structure each section in the same way: we describe the conclusions that have been drawn from previous reviews of the literature, we discuss the behavioural mechanisms that might explain this conclusion or additional behavioural mechanisms that may need to be explored, and we generate some hypotheses that future interventions could test.

## 3 Results

Decision making does not occur within a vacuum. Where we are (the physical environment), what we know (information provision), and who we are with (social context) all influence decisions, particularly ones about health-related behaviours. We discuss physical activity interventions under these three headings as they are the most common targets of interventions.

### 3.1 The environment

We use environment to mean physical structures and amenities that may influence decisions about physical activity. The importance of an appropriate environment has been championed internationally (Department of Health and Ageing, 2013, Institute and for Health and Care Excellence, 2010, Transportation Research Board, 2005).

Interventions that prioritise the regeneration of facilities have produced mixed results. To take two examples, a regeneration programme in the most deprived areas of England designed to tackle health, education, employment, and crime did not increase physical activity levels, despite improvements in other health metrics such as mental health (Beatty et al., 2010). Meanwhile, a study in the Netherlands found positive effects of regeneration on leisure time walking, but not cycling or sports (Kramer et al., 2014). In both cases, physical activity was only one of a range of issues targeted.

Some research has sought to improve specific areas for physical activity within neighbourhoods. Evidence is mixed but weak overall. Positive findings include a five- to nine-fold increase in attendance in low-income areas of San Francisco following renovation of playing fields, with more park visitors engaging in sedentary, moderate and vigorous activities (Tester and Baker, 2009). In Los Angeles, giving park directors a modest budget to spend on raising attendance and marketing the changes, increased usage by 7–12% compared to a control group, although including community feedback in the process made no additional difference (Cohen et al., 2013). Contrastingly, other studies report no effect of regenerating park facilities. One even recorded a reduction of 25% in park use and physical activity, partly attributed to an accompanying drop in scheduled and organised activities (Cohen et al., 2009). A follow-up found that providing classes and frequent user programmes alone were insufficient to increase park use and physical activity (Cohen et al., 2017).

The walkability of neighbourhoods is another target of environmental interventions. Poor physical design was consistently cited as a barrier to activity in one review, particularly walking (Kramer et al., 2017). The most common flaws were absence of available settings, safety concerns, inconvenience of location, lack of amenities, and lack of aesthetic appeal. Another review assessed how ‘smart growth’ principles in urban design affect physical activity levels (Durand et al., 2011). A third examined how the physical and social environment correlate with physical activity (Sawyer et al., 2017). Most of the studies covered are qualitative or cross-sectional, making it difficult to determine causality. Nevertheless, we describe two studies below because they may provide specific behavioural insights.

A cross-neighbourhood comparison found that the walkability of a location has a bigger impact on how much people exercise in areas of high crime compared to in areas of low crime (Bracy et al., 2014). Similarly, when people feel socially connected to their neighbourhood *and* perceive it to be highly walkable they display greater levels of recreational and transport-related activity, compared to peers who either feel socially connected *or* perceive their environment to be highly walkable (Kaczynski and Glover, 2012).

Together, the cross-sectional and intervention studies investigating regeneration of the environment suggest two conclusions. First, while a suitable environment may be a prerequisite for physical activity, the evidence for interventions that focus purely on environmental regeneration is weak. A recent review of urban green space interventions (not solely in disadvantaged populations) concluded that environmental changes alone were unlikely to enhance physical activity, unless awareness raising was also undertaken (Hunter et al., 2019). The broader behavioural science literature may help to understand this conclusion. The large body of recent work on ‘nudging’ suggests that the environment can be an important and often subtle influencer of behaviour (Thaler and Sunstein, 2009). A ‘nudge’ is a change to the environment that alters people’s behaviour without forcing them to change or changing material incentives (Thaler and Sunstein, 2009). Only a subset of nudges are effective. The Behavioural Insights Team in the United Kingdom suggest that interventions must make it easy, attractive, social and timely if they are to alter behaviour (Service et al., 2014). Easy means making the desirable option hassle-free and simple, attractive means drawing attention and rewarding behaviour change, social means considering group influences, not just the individual, and timely means presenting a desirable option when someone is most likely to be receptive. In the context of environmental regeneration schemes, a regeneration project may successfully upgrade facilities but not increase physical activity because it does not sufficiently draw the attention of the target community and make participation an easy, attractive, and social experience. We are not aware of studies that have systematically tested these other potentially important factors within intervention designs.

Our second conclusion is that regeneration schemes might benefit from greater community involvement. ‘Place attachment’ is the bond that individuals make with their environment (Scannell and Gifford, 2010). People who are more attached to their neighbourhood perceive fewer risks in it, even where risks occur, and show greater environmentally-friendly behaviours (Scannell and Gifford, 2010). Social connectedness to one’s neighbourhood is correlated with greater physical activity (Kaczynski and Glover, 2012). While place attachment alone is unlikely to influence physical activity directly, it may contribute to how people respond to regeneration schemes. As the research is mostly cross-sectional, we can’t say if increasing place attachment would enhance the efficacy of regeneration schemes or vice versa, but it may be worth testing. Other work in behavioural science indicates that being involved in the creation of something, or perceiving a sense of ownership over it, increases the value people place on it (Norton et al., 2012, Thaler, 1980). We do not know if this extends to regeneration schemes for physical activity, but future research could test this. Very few interventions test the effect of community involvement (Stasi et al., 2019). One study that tested regeneration with and without community involvement did not find an effect, but also noted that involvement was patchy and there may have been contamination between groups (Cohen et al., 2013). Provision of feedback alone is different from creation and inducing a sense of ownership. As other studies have not directly compared interventions with and without community input, we can only propose this is an element worth exploring.

### 3.2 Information provision

Providing information is one of the most common intervention for health-related behavioural issues (Bull et al., 2018, Cleland et al., 2012, Lehne and Bolte, 2017, Michie et al., 2009). Some suggest that disadvantaged groups benefit more from this type of intervention because they may start with a knowledge deficit (Michie et al., 2009). Yet evidence for an effect of information provision alone is inconclusive or weak (e.g., Cleland et al., 2012, Michie et al., 2009). In a systematic review, eight studies produced only small or no impacts on physical activity, despite educational campaigns that targeted lifestyle change strategies, problem-solving skills, knowledge of available resources, and skills training (Cleland et al., 2012). Given the diversity of methods and lack of detail about them, most reviews have not been able to carry out granular analyses of successful components of information interventions (Craike et al., 2018). However, the broader conclusions made by the reviews offer some behavioural insight.

#### 3.2.1 Multi vs single behaviour interventions

Interventions are generally more effective when focussed solely on physical activity rather than on multiple health behaviours (e.g., healthy eating and physical activity) (Bull et al., 2018, Craike et al., 2018). Some have suggested that this may be because participants taking part in multi-behaviour interventions may be primarily interested in weight loss and so the changes to dietary intake have more immediate results than changes to physical activity (Bull et al., 2018). Others suggest that multi-behaviour interventions may have used different methods to single behaviour interventions, may be less intensively focussed on physical activity as an outcome, or may be more difficult for participants to follow because of the multiple changes required (Craike et al., 2018).

A behavioural science interpretation is that single and multi-behaviour interventions may focus on different goals, and that this may influence motivation. Having a goal is a strong motivator of behaviour change, but having too many goals or focussing on too many behavioural changes at once can be stressful and de-motivating (Craike et al., 2018, Hallworth et al., 2016, Latham and Locke, 2006, Swann et al., 2021). Secondly, multi-behaviour interventions may be less successful because they require a persuasive rationale for each behaviour that is targeted. The Presenter’s Paradox describes the phenomenon whereby strong arguments for something are paradoxically weakened by the addition of less strong arguments, even though the total number of strong arguments remains the same (Weaver et al., 2012). In an example of this, one study gave students either a short list of strong reasons to exercise or a longer list containing the strong reasons to exercise and some additional less strong reasons. Those who only saw the combination of strong and less strong reasons had less interest in exercising afterwards than those exposed to the list of fewer strong reasons (Weaver et al., 2016). Many information campaigns rely on “Top ten reasons to…” or variants on this under the mistaken assumption that adding reasons must increase persuasiveness (Weaver et al., 2016). Yet if not all arguments for something are equally persuasive, the weaker arguments may decrease persuasiveness of the stronger ones. It is possible that multi-behaviour interventions fail when the rationales for carrying out the multiple behaviours required are not equally persuasive. It is important to add, as has been noted before, that these conclusions about single versus multi-behaviour interventions are made from comparing different studies (Craike et al., 2018). An important direction for future research will be to test differences in targeting single versus multiple behaviours within a single study. Secondly, interventions could consider what rationales are given for exercise. The Presenter’s Paradox study above investigated intention to exercise rather than exercise itself. Future work could assess whether giving people one strong rationale for exercising is more effective than giving multiple rationales on actual levels of physical activity. Finally, as individuals may be persuaded by different rationales for behaviour change then asking people to pick their own strongest rationale for exercising and reminding them of this throughout an intervention may be worth testing.

#### 3.2.2 Feedback

Another conclusion from reviews of physical activity interventions is that providing information on the antecedents of exercise can decrease effectiveness, while providing an opportunity to practice exercise as part of the intervention can increase effectiveness (Bull et al., 2018). Applying a behavioural science lens to these two conclusions, we suggest that they may be related to when people get feedback about their behaviour. Much psychological literature shows that feedback can influence behaviour change but the type, timing and method influences success. Immediate feedback, in the form of a reward, is more effective at changing behaviour than feedback given pre-emptively or too long after (Skinner, 1953, Skinner, 1969). Often, physical activity interventions discuss facilitators of exercise with individuals. However this type of pre-emptive feedback may not be as successful as immediate feedback while exercising. Some studies have combined education with exercise classes or pedometers that provide immediate feedback and these have successfully increased physical activity (Clarke et al., 2007, Hovell et al., 2008). While we cannot be sure that the feedback was the element that determined success in these trials, it is possible that if feedback is seen as a type of reward, the immediacy of that feedback will increase the likelihood of the behaviour being repeated (Cooper et al., 2013, Skinner, 1953, Skinner, 1969). This may in part explain why pedometer-based feedback has been shown to increase physical activity (Bravata et al., 2007, Lehne and Bolte, 2017). The conclusions drawn by previous reviews are not based on studies that have directly compared the timing and types of feedback within one study. Future research could compare interventions with and without feedback and also alter the timing of when feedback is given. Technological advances now allow feedback to be given more easily and automatically through mobile phones and other devices, meaning that timing and type of feedback can be varied and outcomes compared. Note that at least one review has attempted to analyse Just In Time Adaptive Interventions (JITAIs) where feedback on health behaviours is given immediately and adaptively when needed, but found that no study was sufficiently powered to detect effects and so conclusions could not be drawn (Hardeman et al., 2019). Any future work will need to consider sample size and power.

#### 3.2.3 Information content

A third conclusion from reviews of the literature is not about interventions themselves, but about descriptions of them. Information provision interventions have used diverse methods but often without providing sufficient detail to analyse the content (Craike et al., 2018). There is unlikely to be a ‘silver bullet’ phrase that motivates physical activity, but behavioural science has long shown that the framing of information can influence how it is understood, retained and acted on (Tversky and Kahneman, 1981). To give an example, when an abstract challenge is described as a task that most people perform equally well on, but some people perform remarkably well on (an ‘achieve success’ frame), people tend to be more motivated than when the same task is described as one that most people perform equally well, but some people perform remarkably poorly on (an ‘avoid failure’ frame) (Elliot and Harackiewicz, 1996). These findings are consistent with Prospect Theory (Kahneman and Tversky, 1979), which predicts that when preventive behaviours are associated with low risk, promotion is best achieved through positive message framing (Jones et al., 2003). This may be applied to physical activity interventions. Warnings about physical inactivity may be framed as failure (i.e., how many people are failing to be physically active) or as success (i.e., how many people are physically active). A study that tested this found that, physical activity gain-framed messages, such as “Physical activity can improve your health – get active!” were more effective at encouraging activity uptake than loss-framed messages, such as “Physical inactivity can cause health problems – get active!” (Latimer et al., 2010, Latimer et al., 2008). Another study found that gain-framed messages were more successful than loss-framed ones at encouraging inactive people to make action plans, but only for those people who were already worried about failure (Michalovic et al., 2018). The latter finding is important as there may be individual differences in how people respond to different messages and therefore scope for individualising message frames within studies. Message framing may thus either reduce or enhance the effects of any information-based intervention. It would be helpful to establish whether previous physical activity campaigns inadvertently adopted loss-framed approaches rather than gain-framed ones but more detail in procedural descriptions is needed to allow this analysis. Future work could directly test different frames on both motivation and physical activity.

### 3.3 Social context

Arguably one of the strongest findings across reviews of the physical activity literature is that group or community-focused interventions are more effective than individually-targeted ones (Cleland et al., 2012, Cleland et al., 2013, Craike et al., 2018, Taylor et al., 1998). However, a group can be defined in different ways and the efficacy may differ depending on who is in the group, whether the group is the method of delivery, whether the group aspect of the intervention is salient to individual members, whether identification with the group forms part of the intervention itself and how accountable individuals feel to others in the group (for discussion of some of these see Burke et al., 2006). Behavioural science can offer some insight into why each of these aspects of a group may matter and how they could be harnessed to increase the impact of physical activity interventions.

#### 3.3.1 Group saliency

An analysis of the geographic locations, social network ties and daily running patterns of 1.1 million people, worldwide, over a 5-year period showed that exercise is “socially contagious” in terms of how far people run, how long they run for and how fast they run. An individual’s behaviour tends to be influenced by friends who are slightly worse or slightly better than themselves at the activity, and not by those who are far worse or far better (Aral and Nicolaides, 2017). Similarly, analysis of a mobile phone app competition found that walking challenges increased physical activity only if participants competing against each other had similar levels of baseline activity (Shameli et al., 2017). This may be related to “social comparison theory”, which is the tendency for people to evaluate themselves based on peers who are like them (Festinger, 1954). Other work has shown that telling office workers that their peers are physically active has a much greater impact on their own levels of physical activity than telling them about the benefits of physical activity (Priebe and Spink, 2012), although the same effect was not found for university students. Differences in how each group formed their group identity or perceived similarity with peers may account for the difference.

Behavioural science may explain at least part of this effect as group exercise may reformulate a social norm. International data show that members of socially disadvantaged groups are substantially less physically active compared to others (Craike et al., 2018, Lunn, 2007). Hence, a physical activity intervention delivered in a group setting may establish new social norms, as individuals witness previously inactive peers becoming active. Future work could test whether a group intervention that actively focusses attention on the similarities between group members is more effective than one that does not. Going further than this, group interventions could test whether feedback during the intervention about the group’s increasing physical activity could change the social norm and therefore influence individuals’ physical activity.

#### 3.3.2 Group identity

At least one review has shown that while group-based physical activity interventions are more effective than individual ones, interventions that promote group cohesiveness are more successful again (Burke et al., 2006). Behavioural science would predict this because when individuals affiliate with members of a particular group, they tend to incorporate the values of the group into their sense of self and align their attitudes and behaviours to it (Stevens et al., 2017, Tajfel and Turner, 1986). Despite substantial differences between interventions, consistency in achieving positive outcomes appears to be robust (Estabrooks et al., 2012). One intervention that grouped individuals by either age or gender, rather than in mixed age and gender groupings, was more successful at promoting exercise adherence over many months (Beauchamp et al., 2018). A review of interventions among socially disadvantaged women goes so far as to suggest that group delivery should be considered “an essential element of physical activity promotion programs targeting this group” (p. 197 Cleland et al., 2013). Other research shows that people in exercise settings tend to create in-groups, and the opportunity to exercise with members of the in-group predicts subsequent exercise habits (Beauchamp et al., 2018, Bruner et al., 2014). Psychological research shows that it is possible to establish group identity by random allocation of people to groups (Billig and Tajfel, 1973). This suggests that group interventions need not necessarily be based on pre-existing shared characteristics but can create group affiliation. However, there are potentially other aspects of identity that could be more powerful. For example, separating people into groups based on a pre-intervention questionnaire about what they like or what their goals are might increase identity with the group. It may also be possible to foster a group identity throughout an intervention in much the same way that sports teams create a group identity, rather than relying on pre-existing characteristics. Rather than merely carry out an intervention within a group setting, future work could test whether actively increasing group bonding and affiliation throughout the intervention influences success.

#### 3.3.3 Accountability

Although people may recognise that they are part of a group, and feel connected to that group, their behaviour is also likely to be influenced by how accountable they feel. Two types of interventions that are designed to increase accountability are incentives and commitment contracts. While these interventions often engage financial motives, the induced motivation is partly social, because incentives and contracts revolve around agreements with others who observe the individual’s behaviour; they engage psychological forces such as pride, face-saving, and desire for status.

A commitment contract is a promise to do something in the future, such as exercise (Royer et al., 2015). Commitment contracts to be physically active have shown some success in the general population, but these have mostly involved financial commitments where people put their own money up as a stake, to be returned only if they achieve their commitment. This type of intervention has generated long-term increases in physical activity, sometimes even over years (Bhattacharya et al., 2015, Goldhaber-Fiebert et al., 2010, Royer et al., 2015). While commitment contracts in these studies act on the premise that someone will increase activity to avoid loss, others have shown that guaranteed rewards for achieving physical activity goals are also effective at increasing activity (Jeffery et al., 1998, Mitchell et al., 2013). Physical activity has even been sustained among previously inactive participants after incentives are removed (Charness and Gneezy, 2009). The latter finding is important, as the principle criticism of financial incentive interventions is the potential for extrinsic motivation to crowd out intrinsic motivation, resulting in a drop-off (Gneezy et al., 2011). It is possible that the incentives act as a motivator for enough time until a habit is formed and the behaviour itself becomes intrinsically motivating. These studies have not been carried out among socially disadvantaged groups, however, and financial commitment contracts may only be beneficial for those with the financial resources to make them. Despite this, the psychological mechanism involved may be generalizable to non-financial incentives as well. A non-financial commitment contract could involve individuals writing down a commitment for future physical activity that they know will be sent back to them later, thus inducing a similar sense of potential regret that financial commitment contracts achieve. It might also be possible to combine group effects with commitment contracts by having people make commitments to a group they feel accountable to. While giving individual financial incentives to every individual may be too costly for some interventions, it may be possible to combine group accountability with incentives such that the group receives a shared reward - a community item or an event for example - for achieving physical activity goals. These are hypotheses based on the findings that group effects and commitment contracts are effective separately, but future work could test whether an intervention that combined both was more effective again.

## 4 Discussion

Physical inactivity is a major challenge facing policymakers, community leaders and individuals, particularly, but not limited to, socially disadvantaged communities. Literature reviews often suggest that intervention research tends to be of mixed quality and efficacy without clear indications as to what predicts a successful outcome. This paper re-examines some of the literature through a behavioural science lens, linking existing conclusions about the literature to some of the behavioural mechanisms that may be behind them. Understanding the behavioural mechanisms behind successful interventions means we can generate specific hypotheses about what to test next. The aim is to understand if there are ‘active ingredients’ of successful interventions that can be incorporated into future interventions.

There are of course limitations. There is a risk of study bias and the inadvertent exclusion of potentially relevant studies. Our initial attempt at systematically reviewing the literature led us to conclude that this approach would not facilitate progress, as too few existing studies were grounded in a theoretical or mechanistic framework. Most were not set up to assess causal mechanisms, making it difficult to make inferences about efficacy. We settled on a more exploratory review to showcase examples of behavioural mechanisms. However, part of our method was to search for papers using specific behavioural mechanisms that may not have been mentioned in previous reviews. This has likely resulted in some studies being overlooked. We sought to minimise this risk by searching through reference lists of included studies, as well as grey literature. The length of the publishing process also means that many new studies that have not yet been included in reviews of the literature will not have been included in our paper. This was unavoidable as we did not want to draw conclusions about the area from individual studies and instead have focussed on reviews that are, by their nature, published some time after the latest studies. Lastly, we started by reviewing reviews of the literature carried out in socially disadvantaged populations but extended this to studies in the general population where we wanted to highlight specific behavioural mechanisms.Although most behavioural mechanisms are likely to be common across groups, we have been mindful to note where some study findings may not generalise.

An important caveat is that this paper does not describe more general frameworks that describe behaviour and behaviour change. We did not discuss cognitive-behavioural theories, motivation theories or more general frameworks of behaviour. A social ecological perspective argues that behaviour is influenced not just by an individual’s own characteristics but by the interaction between the person and others, organisations, community and policy. Interventions that reinforce the main goal by targeting multiple levels are hypothesised to be more powerful than interventions that tackle only one level. Yet there is a danger in carrying out multilevel interventions as it is not easy to find out which part of the intervention is effective or whether some parts may even have contradictory effects (Weiner et al., 2012). It is therefore important to understand mechanisms before launching into multilevel interventions. Our paper is intended to be quite granular in focus rather than broad for this reason. By analysing candidate psychological mechanisms, we suggest hypotheses for future work rather than interventions for immediate implementation.

## 5 Conclusion

Interventions designed to increase physical activity typically incorporate multiple features into an intervention package, often without consideration of the potential effects of each component. Few are based on theory. In day-to-day terms this is a reasonable approach but there is a dilemma within it - researchers and practitioners want to design effective interventions by doing multilevel interventions, but a multilevel intervention may not be effective if some parts contradict each other. We have generated some hypotheses about individual mechanisms throughout this paper, but we do not try to argue that any one alone will necessarily produce positive change. Instead, we argue for future work to design studies such that the effects of specific behavioural mechanisms can be tested both in isolation and together. Designing studies with this in mind would improve the efficacy of future work on physical activity and enhance the development of effective, scalable public health policies.

## 6 Financial statement

This work was funded by Sport Ireland, the authority tasked with the development of sport in Ireland. The funders had no role in the development, write up or decision to publish this manuscript. The authors have no conflicts of interest, financial or otherwise, to declare.

## Declaration of Competing Interest

The authors declare that they have no known competing financial interests or personal relationships that could have appeared to influence the work reported in this paper.
